# Normalization of RNA-Sequencing Data from Samples with Varying mRNA Levels

**DOI:** 10.1371/journal.pone.0089158

**Published:** 2014-02-25

**Authors:** Håvard Aanes, Cecilia Winata, Lars F. Moen, Olga Østrup, Sinnakaruppan Mathavan, Philippe Collas, Torbjørn Rognes, Peter Aleström

**Affiliations:** 1 BasAM, Norwegian School of Veterinary Science, Oslo, Norway; 2 Stem Cell and Developmental Biology, Genome Institute of Singapore, Singapore, Singapore; 3 Stem Cell Epigenetics Laboratory, Institute of Basic Medical Sciences, Faculty of Medicine, University of Oslo, and Norwegian Center for Stem Cell Research, Oslo, Norway; 4 Department of Informatics, University of Oslo, Oslo, Norway; Inserm U869, France

## Abstract

Methods for normalization of RNA-sequencing gene expression data commonly assume equal total expression between compared samples. In contrast, scenarios of global gene expression shifts are many and increasing. Here we compare the performance of three normalization methods when polyA^+^ RNA content fluctuates significantly during zebrafish early developmental stages. As a benchmark we have used reverse transcription-quantitative PCR. The results show that reads per kilobase per million (RPKM) and trimmed mean of M-values (TMM) normalization systematically leads to biased gene expression estimates. Biological scaling normalization (BSN), designed to handle differences in total expression, showed improved accuracy compared to the two other methods in estimating transcript level dynamics. The results have implications for past and future studies using RNA-sequencing on samples with different levels of total or polyA^+^ RNA.

## Introduction

RNA sequencing (RNA-seq) is frequently used for global gene expression analysis. RNA-seq generates short reads from fragmented RNA molecules and the number of reads is proportional to the abundance and length of the transcripts [1]. However, the read count needs processing to accurately represent the expression status of a particular gene [2]. This processing, referred to as normalization, is defined as removal of systematic experimental bias and technical variation with the aim to improve identification of gene expression changes across conditions [3]. Different normalization strategies have been proposed, most of which assume equal amounts of RNA in each experimental unit. For example, for each cell, embryo or organism only a few transcripts change abundance or changes are balanced out. Among normalization methods published are the well-known “reads per kilobase of transcripts per million mapped reads” (RPKM) [4] and the less frequently used median and quantile normalization methods (reviewed in [2]). Another strategy, presented by Robinson and Oshlack [5], introduces a scaling factor called Trimmed Mean of M-values (TMM), which aims to represent the “global fold-change”. However, application of this method results in samples of similar total expression, which may not be biologically correct.

Although equal global gene expression levels are generally acknowledged as an important assumption in all of the aforementioned methods, it is rarely tested. We recently showed that in zebrafish embryos, approximately 70% of maternal transcripts undergo cytoplasmic polyadenylation prior to onset of zygotic transcription, leading to a 50–70% increase in the retrieved polyA^+^ RNA amounts between the 1-cell stage and the ∼1000-cell stage 3.5 h post-fertilization (hpf) [6]. It was subsequently shown that stimulation with a transcriptional activator (c-Myc) increases total and polyA^+^ RNA levels several fold [7]. Furthermore, cancer cells have been shown to contain more total RNA than normal cells [8] and it is well known that different tissues contain different amounts of RNA; for instance, a comparison of embryonic stem cells and fibroblasts reveals a 5.5-fold difference in mRNA levels [9]. Further, cellular stress can dramatically alter the amount of RNA, as shown for heat-shock treated cells [10]. Thus, both under natural and experimental conditions, the critical assumption of equal expression levels between cell types, disease states or developmental stages is no longer valid. This may, depending on its severity, influence the statistical inference and biological interpretation of the results [10].

To account for this, we recently proposed an approach which uses experimentally measured polyA^+^ RNA amounts as scales to normalize different developmental stages [6]. This method is called biological scaling normalization (BSN). We first estimate the concentration of each transcript:
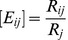
(eq.\ 1)


Where [E_ij_] is the relative abundance of transcript i in sample j, R_ij_ is the number of reads for gene i in sample j, and R_j_ is the total number of reads in sample j. We calculate the average library size:
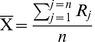
(eq.\ 2)


Where n is the number of samples. A pseudo library size is obtained by multiplying the average library size with a stage specific scaling factor:
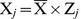
(eq.\ 3)


The scaling factors can be obtained both mathematically and experimentally, as will be demonstrated later. The next step in the normalization procedure is to reassign the pseudo read libraries to each gene based on the previously estimated [E_ij_] value to get the normalized dataset: 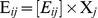
(eq.\ 4)


Similarly to TMM normalization, our method contains a scale which aims at representing change in total expression, also termed the “global fold-change” [5]. However, the TMM scales are derived from the read counts of a trimmed set of investigated transcripts, while the scales used in BSN were based on measurements of polyA^+^ RNA content per embryo [5], [6]. We denote these scales as Z_j_^TMM^ and Z_j_^Bio^, respectively. Importantly, the usage of the scales differs drastically between TMM-normalization and BSN. While during TMM normalization the scale (Z_j_^TMM^) is incorporated during the estimation of transcript concentrations ([E_ij_] = R_ij_/(R_j_*Z_j_^TMM^)), this is done afterwards in BSN (see equation 3). RPKM, as an intermediate approach, normalize without adding any scaling factor ([E_ij_] = R_ij_/R_j_). The rationale behind both RPKM and TMM normalization is similar, and distinct from BSN; they assume little difference in total expression between compared samples. If such a difference exists, it will be reduced after normalization. Rather, BSN seek to retain biological differences between samples, with the assumption that more RNA detected at a particular stage would correspond to more genes being up-regulated.

To test our hypothesis we have validated BSN and compared it to RPKM and TMM normalization under conditions of global increase and decrease of polyA^+^ RNA levels during the first 6 h of zebrafish development. The first 2.5 h are characterized by substantial increase of polyA^+^ RNA [6], while there is massive decay of RNA due to miRNA-430 activation at 3.5 hpf and onwards [11]. Compared to RT-qPCR benchmarks, the results show improved accuracy of expression level changes using BSN compared to the two other normalization methods.

## Results

### Estimation of the scaling factor

A key component in our normalization procedure is the estimation of a reliable measure of global fold-change, denoted Z_j_ throughout this paper. This measure represents the change in total RNA or polyA^+^ RNA, depending on which population is under study. Two methodologies were used to gain an estimate of the fluctuations of RNA levels in the embryo, one biological and one mathematical. First we isolated and measured the amount of total and polyA^+^ RNA from equal numbers of embryos at different developmental time points before zygotic genome activation (ZGA) (1-cell, 4-cell, 16-cell and 128-cell) and after (3.5 hpf and 5.5 hpf). These time periods are referred to as pre- and post-ZGA samples from here on. Total RNA levels did not change significantly between stages, but we did observe a decreasing trend (Fig. S1a in File S1). The levels of polyA^+^ RNA increased from the 1-cell to the 128-cell stage, levelled off towards 3.5 hpf and decreased between 3.5 hpf and 5.3 hpf (Fig. 2; Fig. S1b in File S1). Due to high variance in absolute RNA amounts, we chose to use the polyA^+^ RNA percentage as the normalization scales (Fig. 2) (see methods). We denote these scales as Z_j_^Bio^.

**Figure 1:**
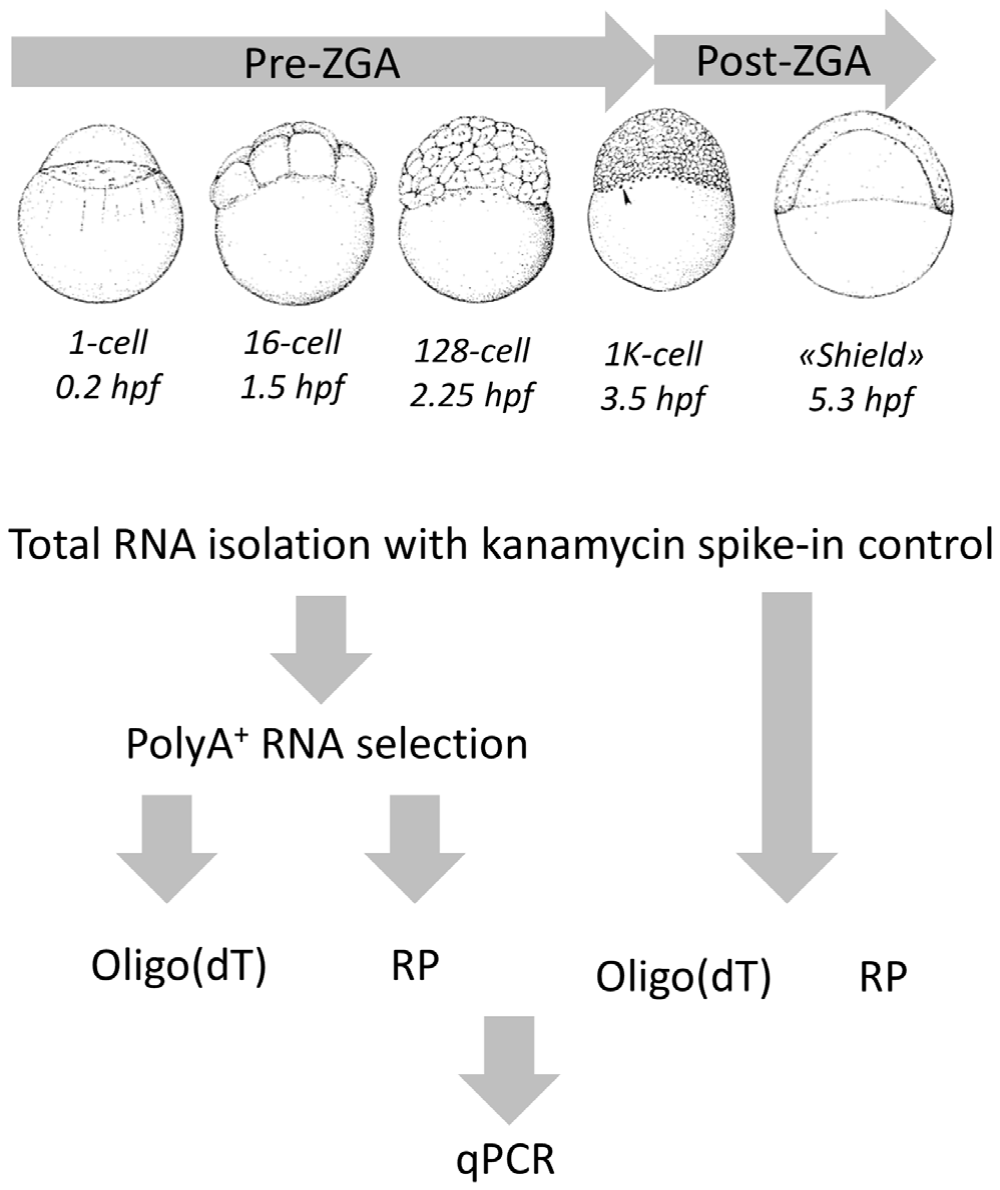
Experimental design. Total RNA from 5 developmental stages pre- and post-ZGA was isolated and kanamycin polyA^+^ RNA was used to adjust for differences in RNA yield. PolyA^+^ RNA was isolated and four cDNA libraries were generated to compare qPCR results using different template and primers.

**Figure 2:**
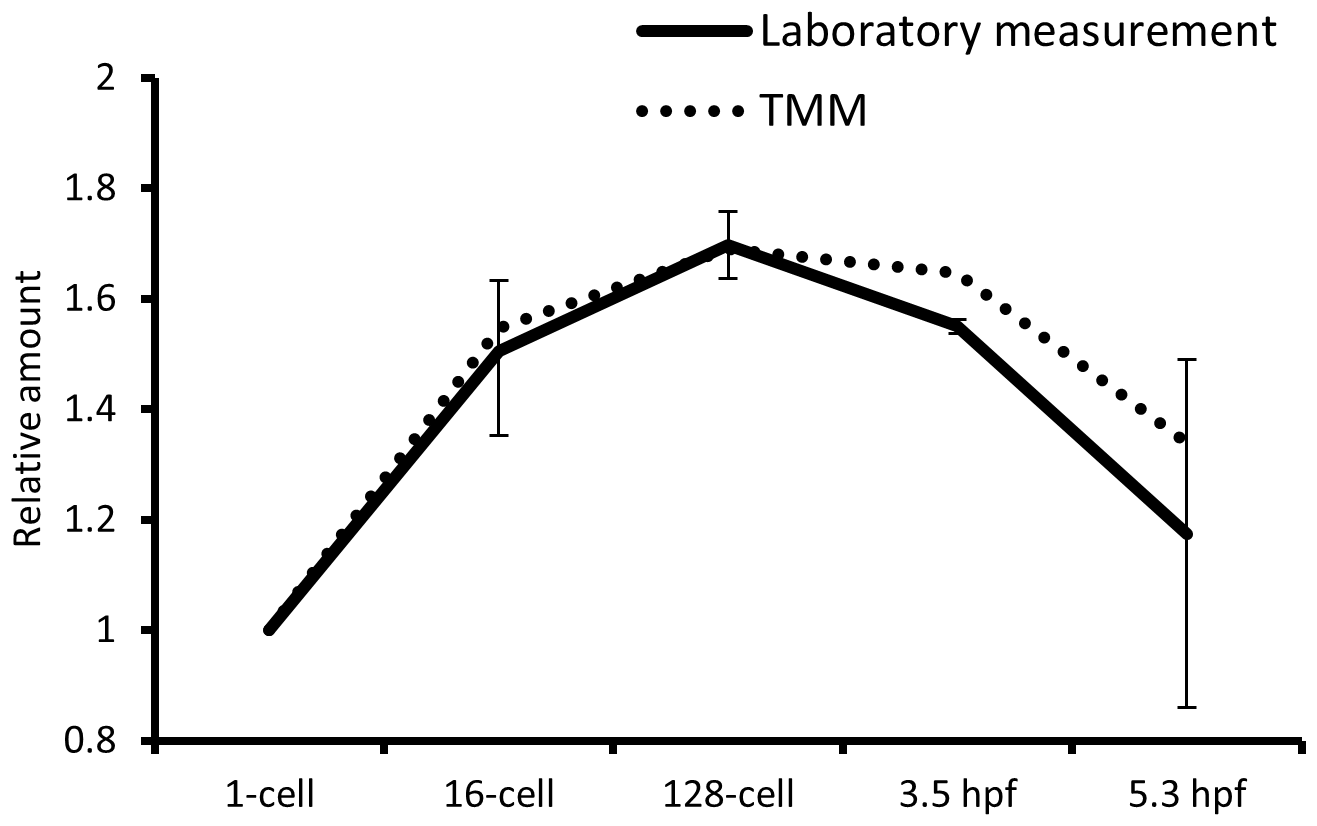
Relative polyA^+^ RNA amounts. Measurements of polyA^+^ RNA determined by a standard laboratory method (full line) and using trimmed mean of M-values (TMM) (stippled line) display an almost identical pattern during early embryogenesis with an early increase and subsequent decrease. The levels are relative to the 1-cell stage.

In a second approach we estimated TMM scaling factors as described by Robinson and Oshlack (2010) (see methods). We denote these scaling factors as Z_j_^TMM^. The Z_j_^TMM^ values correlated well with the Z_j_^Bio^ scales obtained through experimental polyA^+^ RNA measurements (Fig. 2). Moreover, comparison of Z_j_^TMM^ between two different RNA-seq datasets (dataset 1; [6], dataset 2; [12]) showed reproducibility across platforms (SOLiD3 and Illumina), as well as replicates (dataset 2) (Fig. S2a,b in File S1). Also, RNA-seq data derived from total RNA showed no increase pre-ZGA (Fig. S2c in File S1). From these data, we conclude that there are substantial fluctuations in polyA^+^ RNA amounts during development, and that Z_j_^TMM^ and Z_j_^Bio^ are valid estimators of global fold-change under the circumstances studied here. In the remainder of our study, we used the laboratory-derived factor Z_j_^Bio^ for dataset 1 and the Z_j_^TMM^ scales for dataset 2 when normalizing with BSN. We previously square-root transformed the scaling factors [6], however the new analysis shows that this conservative approach is less accurate than using the scaling factors without transformation (Fig. S3a-c in File S1).

### Comparison of total RNA and polyA^+^ RNA derived cDNA libraries reveal fundamental differences

**Figure 3:**
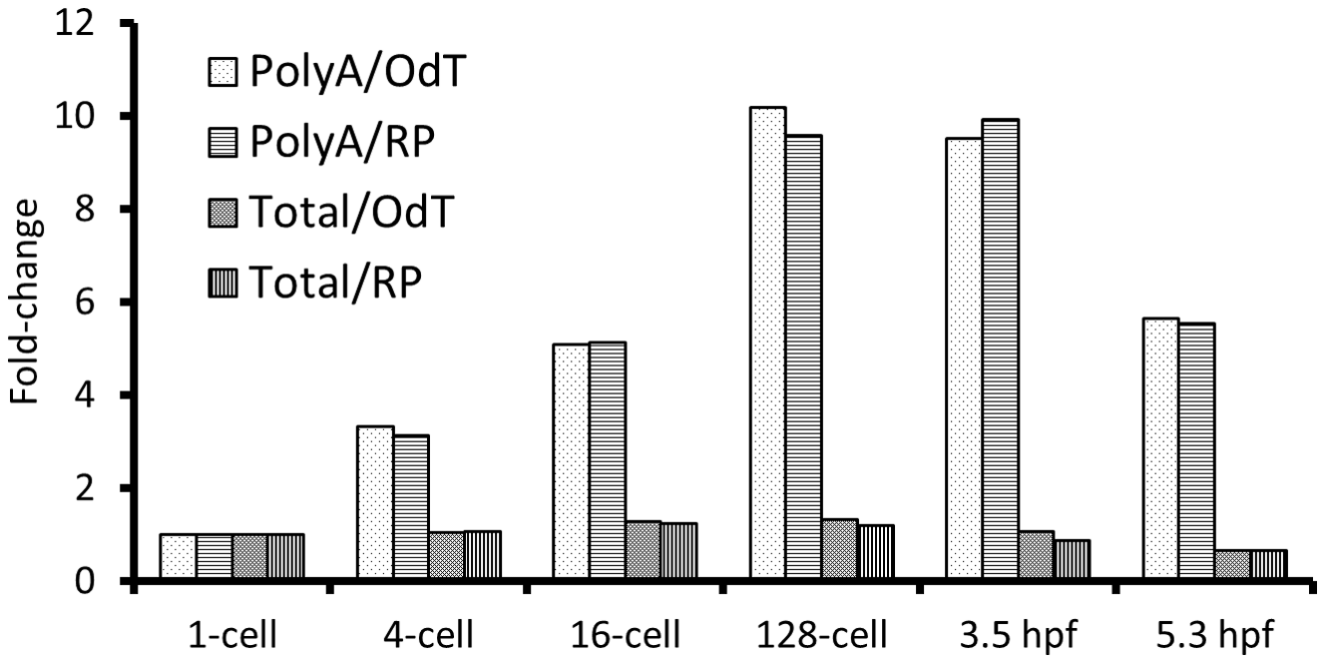
cDNA template and primer comparison. Comparison of RT-qPCR results based on polyA^+^ and total RNA and oligo(dT) and random primers for *stat3*. The increase pre-ZGA is only detected in the polyA^+^ RNA-based cDNA libraries. PolyA  =  polyA^+^ RNA, Total  =  total RNA, OdT  =  oligo(dT) primers, RP  =  random primers.

To determine whether RT-qPCR results are affected by the use of total or polyA^+^ RNA and/or type of primers used to generate cDNA (random or oligo(dT) primers), we performed parallel experiments of the same samples using different combinations of template and primers. The results demonstrate that detection of increase in mRNA abundance pre-ZGA depends on an enrichment of transcripts in the polyA^+^ RNA fraction rather than in total RNA (Fig. 3; Fig. S4a and b in File S1). These results are consistent with an increased polyA tail length of existing transcripts and not *de novo* transcription during the pre-ZGA period [6]. The level of increase post-ZGA is more similar between total and polyA^+^ RNA libraries (Fig. S4c in File S1).

### Comparison of RNA-seq normalization methods

**Figure 4:**
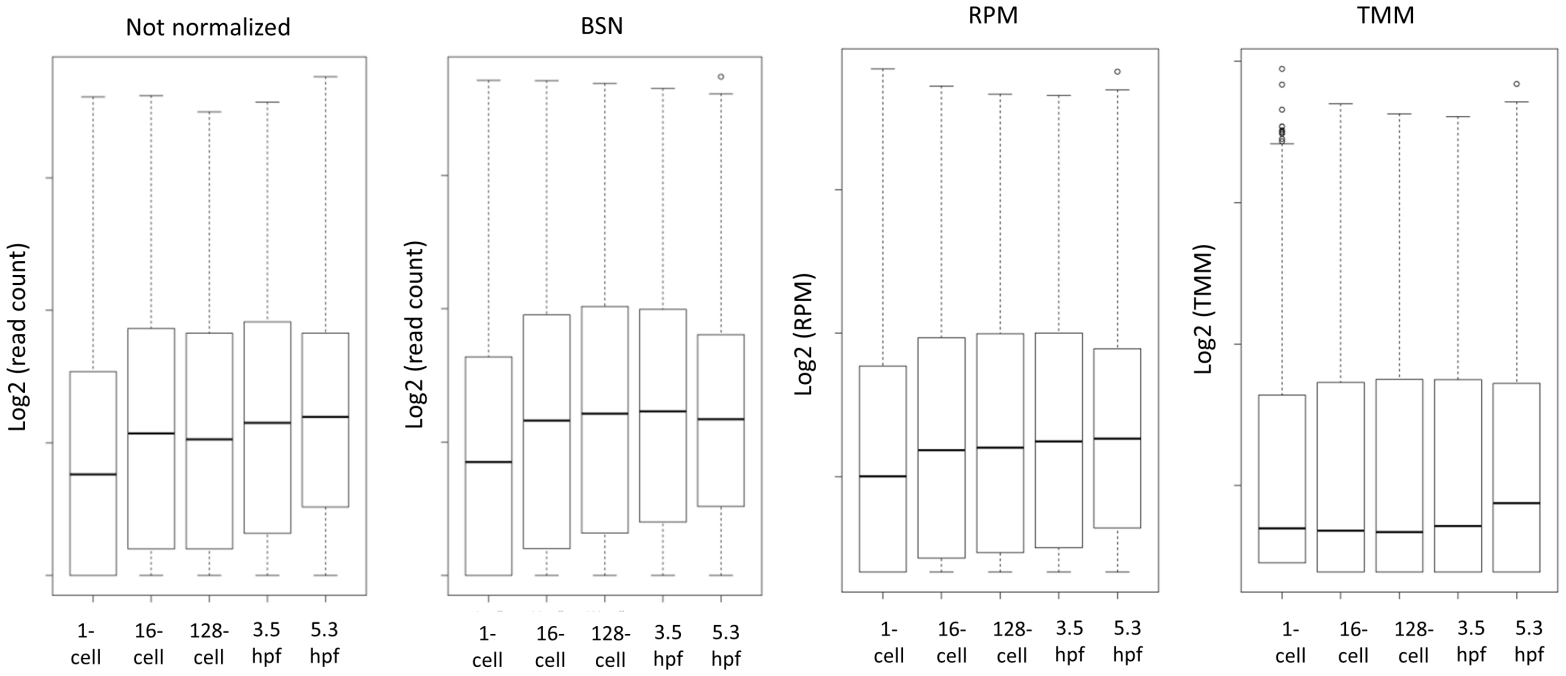
Distribution of gene expression values. Box plot of distribution of transcript counts or values before (not normalized) and after normalization (BSN, RPM and TMM).

Three different methods for normalization of RNA-seq data were compared. Raw read counts were divided by the total number of million mapped reads in each sample as described for RPKM [4], but without dividing by the length of the transcripts; this approach is from here on called Reads Per Million (RPM). TMM normalized values were obtained using the R package “limma” (see methods) and BSN normalized values using Excel (see methods). These three normalization methods represent the main groups of RNA-seq normalization methods available today [2]. The global effect of normalization can be viewed in box plots (Fig. 4). BSN mimics the global polyA^+^ RNA trends (Fig. 2), in contrast to RPM and TMM normalization which cause the samples to become more similar. This illustrates the key difference between the normalization methods compared; BSN seek to maintain biological differences, while RPM and TMM lead to samples with similar distribution of the gene expression levels.

**Figure 5:**
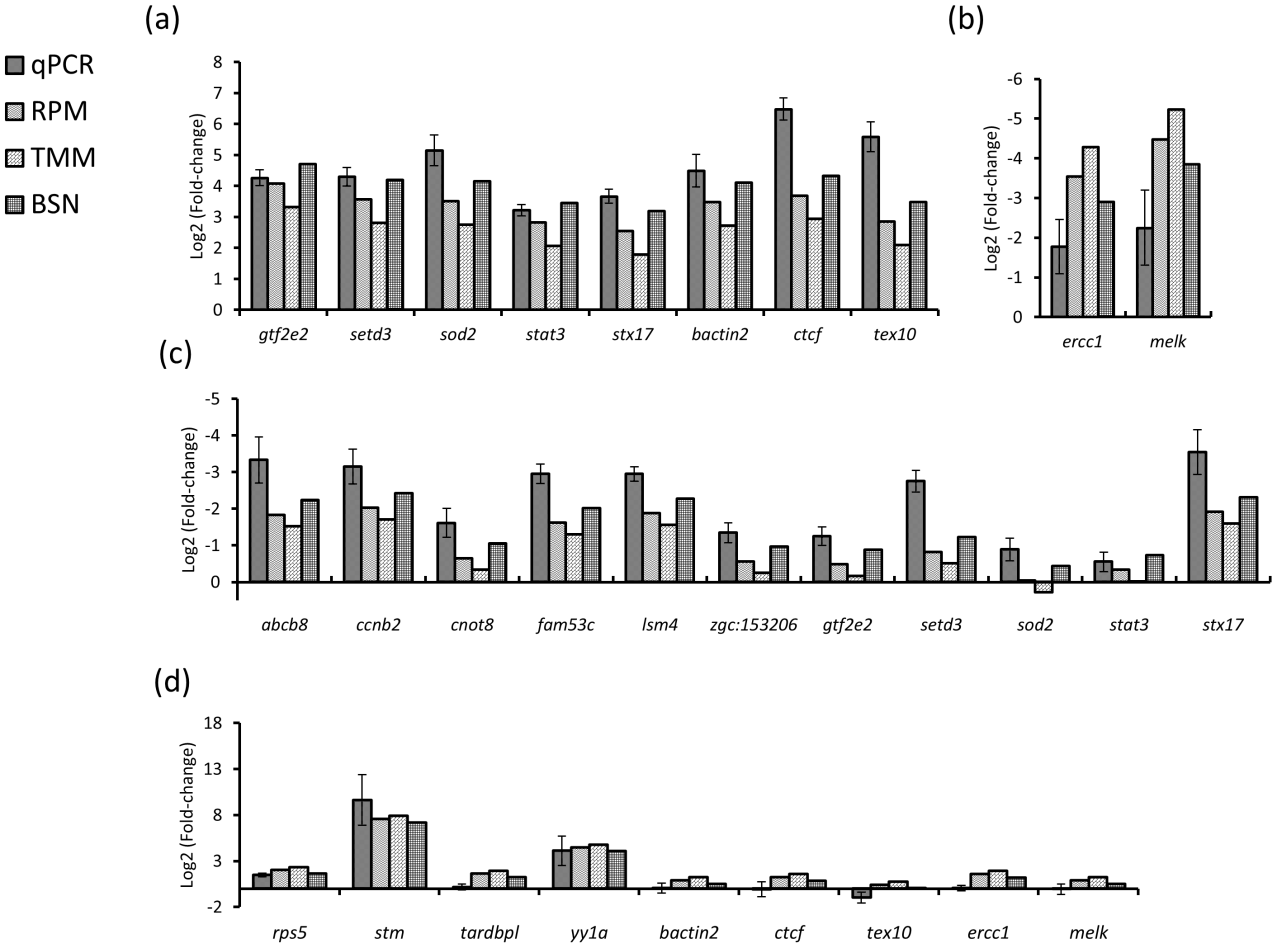
Comparison of normalization methods. Log2-tranformed fold-changes comparing RT-qPCR and RNA-seq data normalized using RPM, TMM and BSN for transcripts increasing pre-ZGA (a), decreasing pre-ZGA (b), decreasing post-ZGA (c) and increasing post-ZGA (d).

At the individual transcript level, we focused on two time points of dynamic change; between the 1-cell and the 3.5 hpf stage (pre-ZGA), and between 3.5 hpf and 5.3 hpf (post-ZGA). Spike-in RNA was added to the Trizol reagent before RNA isolation and polyA^+^ RNA was extracted from equal volumes of total RNA from each stage to ensure unbiased RT-qPCR values for 20 different transcripts (see methods). Transcripts with an increase during pre-ZGA stages (n = 8) are in 7 of 8 examples best approximated by BSN (Fig. 5a). Overall, the pre-ZGA fold changes were 55% and 163% higher for BSN compared to RPM and TMM, respectively. Importantly, two transcripts that decrease pre-ZGA were also best estimated using BSN (Fig. 5b). For all 11 transcripts examined with decreasing expression between 3.5 and 5.3 hpf, the BSN estimated values are in all cases closest to the qPCR results (Fig. 5c). For *sod2*, there is even a difference in the direction of the estimated fold change between the BSN and TMM normalized values. For transcripts examined with an increase from 3.5 to 5.3 hpf (n = 9), the BSN values are closest to the qPCR benchmark in all cases (Fig. 5d). For some of these transcripts we detect substantial differences between qPCR and RNA-seq results (*tardbpl*, *bact2*, *tex10*, *ctcf*); however this is independent of normalization method. On average, the fold changes post-ZGA were 32% and 64% lower for BSN, compared to RPM and TMM. The BSN method also performed best when primer efficiency calculations were used for adjusting fold-changes, and discrepancies between qPCR and RNA-seq were reduced (Fig. S5 in File S1). Taken together our results demonstrate a substantial increase in accuracy using BSN compared to RPM and TMM normalization.

Comparisons between normalization methods and qPCR for transcripts varying post-ZGA using dataset 2 revealed the same trend as for dataset 1; however, there was less discrepancy between qPCR and RNA-seq results (Fig. S6a,b in File S1). BSN normalized expression levels were closest to qPCR values in 10 of 11 examples for down-regulated transcripts, and in all cases for up-regulated transcripts.

## Discussion

Fluctuation in polyA^+^ RNA levels during development is well documented [6], [13]–[15], and the challenge this poses on the accurate representation of expression levels has been met with normalization methods for qPCR and microarray analysis [10], [16]. Similarly, BSN was designed to normalize RNA-seq data that contain global shifts in RNA population sizes [6]. We now demonstrate a clear advantage of using an approach mimicking the polyA^+^ RNA levels (BSN), compared to methods aimed at making samples similar (RPM and TMM normalization). Our results show that the normalized expression values were consistently best approximated by BSN when compared to a RT-qPCR benchmark. Only a few transcripts increasing prior to ZGA were best approximated by RPM normalized values; TMM-normalized values on the other hand were in all cases the least accurate. As expected, both the TMM and RPM normalization strategies leads to underestimation during the increase (pre-ZGA) and overestimation during the decrease (post-ZGA) of polyA^+^ RNA amount, RPM less so than TMM normalization [10], [17]. The choice of normalization method has a profound effect on gene expression estimates; pre-ZGA the BSN normalized fold-changes were 55% and 162% higher than RPM and TMM, while TMM and RPM fold-changes were 64% and 32% higher than BSN post-ZGA. These results call for awareness in the selection of normalization method.

Despite low accuracy of TMM normalization under the circumstances studied here, the TMM scaling factor, Z_i_^TMM^, is a good estimator of global fold change, with high correlation to laboratory measurements. It could therefore be tempting to rely solely on this cost-effective and efficient mathematical approach. However, we strongly encourage laboratory validation of the estimated global fold-changes since the TMM method does not always detect global changes. In the aforementioned example of a global increase in expression after c-Myc stimulation [7], the TMM approach was not able to estimate the increased expression level (data not shown), since all genes were up-regulated. In this case, a strategy with RNA spike-in controls and estimated scaling factors based on local regression (LOESS) was used [18]. Although this spike-in RNA approach may solve the problem of RNA fluctuations in the samples being studied, such a strategy is not useful for interpretation of previously published data where spike-in RNA is not added. In addition, regression analyses are in general sensitive to outliers; in the case of LOESS, this is particularly relevant due to the few data points each local coefficient depends on (96 different RNAs in total). This was recently made relevant by a report on the variability of spike-in controls [19]. However, these concerns must be tested and regardless of outcome, spike-in RNA normalization is a leap in the right direction for datasets comparing samples which differ substantially in RNA amount.

We have shown improved accuracy of normalizing gene expression data using BSN compared to RPM and TMM during developmental stages that display global increases and decreases in mRNA content. Application areas of this approach are expected to be numerous, ranging from comparing gene expression during development, disease, and tissue- and cell-type specification. More generally, our results should be a strong argument for testing the assumption of equal RNA levels in all gene expression dynamics studies.

## Methods

### Ethics Statement

All studies were performed in accordance with animal experiment legislation and guidelines. In Norway zebrafish embryos are not regulated prior to they start feeding at day five post-fertilization. Consequently, no approval was needed for experiments in this study of embryos up till 6 hours post fertilization.

### Embryo collection and RNA isolation

Wild-type zebrafish from AB background were maintained in the zebrafish facility of the Institute of Molecular and Cell Biology, Singapore. Embryos were grown in embryo medium at 28°C and staged according to standard morphological criteria [20]. Total RNA from 100 embryos at each developmental stage (Fig. 1) was isolated using Trizol (Invitrogen, USA) from stages before zygotic genome activation (ZGA) (1-cell, 4-cell, 16-cell and 128-cell) and after (3.5 hpf and 5.5 hpf). Spike-in RNA (polyadenylated kanamycin RNA #C1381, Promega, USA) was added to the Trizol with a final concentration of 0.25 ng/ml. PolyA^+^ RNA was extracted from equal volumes of total RNA from each stage using the MicroPoly(A)Purist™ Kit (Ambion, USA). The amount of total and polyA^+^ RNA was measured with a NanoDrop 2000 (Thermo Fisher Scientific, MA, USA) and a Qubit® RNA Assay Kit (Invitrogen,USA), respectively. RNA integrity was measured using the Agilent RNA 6000 Nano chip on a Bioanalyzer 2100 (Agilent, USA).

### Reverse transcription (RT)-qPCR

mRNA levels for 20 transcripts at each developmental stage were measured by RT-qPCR. The transcripts were chosen systematically to represent different expression patterns. We used an equal number of embryos used synthetic kanamycin RNA to adjust for differences in RNA yield [10], [16], [17]. Equal volumes of samples across all developmental stages were used in polyA^+^ RNA extraction as well as in RT and qPCR reactions. For cDNA synthesis we used Superscript III First Strand Synthesis System (Invitrogen, USA). For a subset of transcripts, we performed RT on total and polyA^+^ RNA using either oligo(dT)_20_ or random hexamer primers, and generated four groups of cDNA: (1) total RNA + oligo(dT), (2) total RNA + random primers, (3) polyA^+^ RNA +oligo(dT), (3) polyA^+^ RNA + random primers (Fig. 1). For the rest of the transcripts we focused on four stages (1-cell, 16-cell, ZGA and post-ZGA) and used only oligo(dT)_20_ primers and polyA^+^ selected RNA. qPCR was performed (primers listed in Table S1 in File S1) using SYBR green (Fermentas, Lithuania). Cycle threshold (Ct) values were normalized against the kanamycin spike-in control and 2^−ΔΔCt^ values were obtained as described [21]. Primer efficiency calibrated values were calculated according to [22] (E_target_^Δct target (ctl-sample)^/E_ref_^Δct ref (ctl-sample)^). The fold-change values were log2-transformed.

### RNA-sequencing and normalization

We used RNA-seq data from two different previously published studies for comparison of normalization methods. The first dataset (“dataset 1”) was generated using SOLiD3 technology [6] (GEO accession number GSE22830). The reads were strand specific and single end, generated from five different stages, all overlapping the qPCR data (1-cell, 16-cell, 128-cell, 3.5 hpf and 5.3 hpf). The second dataset (“dataset 2”) [12] was downloaded from GEO (GSE32898) and mapped with Tophat [23]. The dataset 2 RNA was collected from 2/4-cell stage, 3 hpf, 4.5 hpf and 6 hpf embryos. The 3 and 6 hpf samples in dataset 2 were compared to qPCR results from 3.5 and 5.3 hpf embryos. Both datasets were generated from polyA^+^ selected RNA. Dataset 1 using Poly(A) Purist kit (Ambion, USA) and dataset 2 using PolyA purist-MAG-kit (Ambion). The total RNA starting material was in both cases extracted with Trizol (Invitrogen). For comparison of TMM values we also downloaded a dataset based on total RNA [24]. Counting of reads per gene was performed using HTSeq (http://www-huber.embl.de/users/anders/HTSeq/).

The raw counts were normalized against the total number of mapped reads per million (RPM), to represent RPKM [4]. TMM-normalization was performed by calculation of Z_j_^TMM^ scaling factors using the R package “edgeR” and the “calcNormFactors” command [5]. These were used in library size calculations in the “voom” command in the R package “limma” and TMM-normalized expression values were retrieved from the “E” slot in the returned object. These log2-transformed values were used to calculate fold-change through subtraction. BSN normalized values were obtained as described in equation 1-4 using Excel. We obtained two sets of scaling factors: i) Mathematical based scales, Z_j_^TMM^, were obtained as for TMM-normalization (using “edgeR” with the command “calcNormFactors” with default settings) [5]. ii) Biological scales, Z_j_^Bio^, were obtained through PolyA^+^ RNA laboratory measurements and calculation of the percentage polyA^+^ RNA of the total RNA. Each stage specific percentage was then divided by the 1-cell stage percentage to generate the Z_j_^Bio^ scales. Replicates in dataset 2 were first normalized within each group through multiplying concentrations (eq. 1) with the average library size for each group. The average library size across all stages were then calculated (eq. 2). Scaling factors were obtained using “edgeR” and the command “calcNormFactors” on a set of replicate averaged values. These scales were used to obtain stage specific library sizes (eq. 3). Concentrations of the transcripts were then again calculated and multiplied with the stage-specific library size (eq. 4). The resulting normalized counts all had correlation coefficients >0.99. All raw and normalized values are available in dataset S1 and S2.

### Comparison between RT-qPCR and RNA-seq

For comparison of normalization methods we selected transcripts with a dynamic expression pattern. Fold-changes for two different time points were calculated depending on whether transcripts expression levels changed or not: i) from the 1-cell to the 3.5 hpf stage (“pre-ZGA”) and/or ii) from 3.5 to 5.3 hpf (“post-ZGA”). Fold changes were calculated as the ratio of 3.5 hpf to 1-cell and as the ratio of 5.3 hpf to 3.5 hpf and subsequently log2-transformed.

## Supporting Information

File S1 **Table S1 and Figures S1–S6.** Table S1. List of primers. Forward and reverse primers used for qPCR. Figure S1. Changes in total and polyA^+^ RNA during development. a) Amount of total RNA per embryo at different developmental stages. b) Amount of polyA^+^ RNA per 100 embryos at different developmental stages. Vertical bars represent standard errors. Figure S2. The TMM scaling factor. a) The TMM scaling factor estimated using dataset 1 and 2. We observe very similar values. b) The TMM scaling factor obtained using the replicates in dataset 2. The TMM values are very reproducible. c) The TMM scale factor when RNA-seq data based on total RNA was used. Figure S3. Comparison of scales. We either square-root transformed or used that scales directly and compared the normalized fold-changes to RT-qPCR results. a) Transcripts with dynamic change pre-ZGA. b) Transcripts with decreased abundance post-ZGA. c) Transcripts with increased expression post-ZGA. Vertical bars represent standard deviations. Figure S4. Comparison of RT-qPCR results depending on RNA template (total or poly^+^ RNA) and primers (random or oligo(dT) primers) for *setd3* (a), *gtf2e2* (b) and *yy1a* (c). The increase pre-ZGA is dependent on template (*setd3* and *gtf2e2*) and not primer type. Figure S5. Efficiency calibrated fold-changes for a subset of transcripts. Vertical bars represent standard deviations. Figure S6. Comparison normalization methods using dataset 2 for transcripts with decreased expression post-ZGA (a) and increased expression post-ZGA (b). Vertical bars represent standard deviations.
(PDF)
