# Supplementary material for: Normalization of RNA-Sequencing Data from Samples with Varying mRNA Levels
Source: PLoS One. 2014 Feb 25;9(2):e89158. doi: 10.1371/journal.pone.0089158 (PMC3934880; doi:10.1371/journal.pone.0089158)
Supplement: File S1 — Table S1 and Figures S1–S6. Table S1. List of primers. Forward and reverse primers used for qPCR. Figure S1. Changes in total and polyA+ RNA during development. a) Amount of total RNA per embryo at different developmental stages. b) Amount of polyA+ RNA per 100 embryos at different developmental stages. Vertical bars represent standard errors. Figure S2. The TMM scaling factor. a) The TMM scaling factor estimated using dataset 1 and 2. We observe very similar values. b) The TMM scaling factor obtained using the replicates in dataset 2. The TMM values are very reproducible. c) The TMM scale factor when RNA-seq data based on total RNA was used. Figure S3. Comparison of scales. We either square-root transformed or used that scales directly and compared the normalized fold-changes to RT-qPCR results. a) Transcripts with dynamic change pre-ZGA. b) Transcripts with decreased abundance post-ZGA. c) Transcripts with increased expression post-ZGA. Vertical bars represent standard deviations. Figure S4. Comparison of RT-qPCR results depending on RNA template (total or poly+ RNA) and primers (random or oligo(dT) primers) for setd3 (a), gtf2e2 (b) and yy1a (c). The increase pre-ZGA is dependent on template (setd3 and gtf2e2) and not primer type. Figure S5. Efficiency calibrated fold-changes for a subset of transcripts. Vertical bars represent standard deviations. Figure S6. Comparison normalization methods using dataset 2 for transcripts with decreased expression post-ZGA (a) and increased expression post-ZGA (b). Vertical bars represent standard deviations. (PDF) [file pone.0089158.s001.pdf]

Supplemental Table 1. List of primers. Forward and reverse primers used for qPCR.

**RefSeq**	**Name**	**forward**	**reverse**
NM_181601	*bactin2*	TGCGGAATATCATCTGCTTGTAAC	GAGCATCATCTCCAGCGAATC
NM_001001844	*ctcf*	CATTGCCTGAGGGCTTTCAG	CGTCTGTGTCGCACCAGCTC
NM_212705	*tex10*	ACCACACACAGGCATCTGGAC	GTAGGGTTAGCAGACAGCAACTCC
NM_001007449	*stx17*	CACATCAACTCCAGCCGCAC	CAACACTACGAACTCGTCCACAC
NM_199976	*sod2*	GGACAAATCTGTCACCCAATGG	CAGCCTGAGCCCTGAACAGC
NM_200696	*lsm4*	AGAGGAGGTGGACCTGGAAGAG	GCTTTTTGTCTTGTTGCTGTCC
NM_213479	*cnot8*	TCAAGTGGCTCTCCTTTCACAG	CGTGTTCTTCCTCTGGCAGC
NM_001076755	*zgc:153206*	CTTGGTTCTTGGTGGCACAGC	TGGACGATGTAACGGCACATG
NM_199430	*ccnb2*	CATCATGGAAATGCACGCTC	CTGCACCGCTTACTGGTCCAC
NM_001017544	*abcb8*	CTGTCCTGTGGGAGTTTGTTCG	CCAGATCCCCCAACATTAGAGG
NM_001111239	*fam53c*	CTGCTGATGGATTGATGCCTG	GGGAAGCTGGAGCTGAGCAC
NM_198817	*stm*	CCTAGCAACGAGGACTCAAGAAC	GGACGTGAAACTCTGGTTATCTTG
NM_131177	*jup*	CCTACACCACCACATTCACAGAG	CATCACAGTCTCAGGAAACATGG
NM_200852	*rps9*	CCTAGCAGCAATGTTAGCCTGAG	GTACGTCTTACTGCAAACCCAAG
NM_173232	*rps5*	AGCTCATCAACGCTGCCAAG	ACAAAATGGAGATTAGCGGTTAGAC
NM_198364	*tardbpl*	GGGGCAATCTGGTGTACGTG	CAAATCTGATGTCTTCTGGATGC
NM_206888	*melk*	CAAGGTTATGCCCATCGAGAC	TATGCAGGACTGCCACAACATG
NM_001103138	*ercc1*	GATTCAGCGTTCTCAAAGGACAG	AACAAACTCTGCATAGGACAGTGG

Fig. S1

(a)

(b)

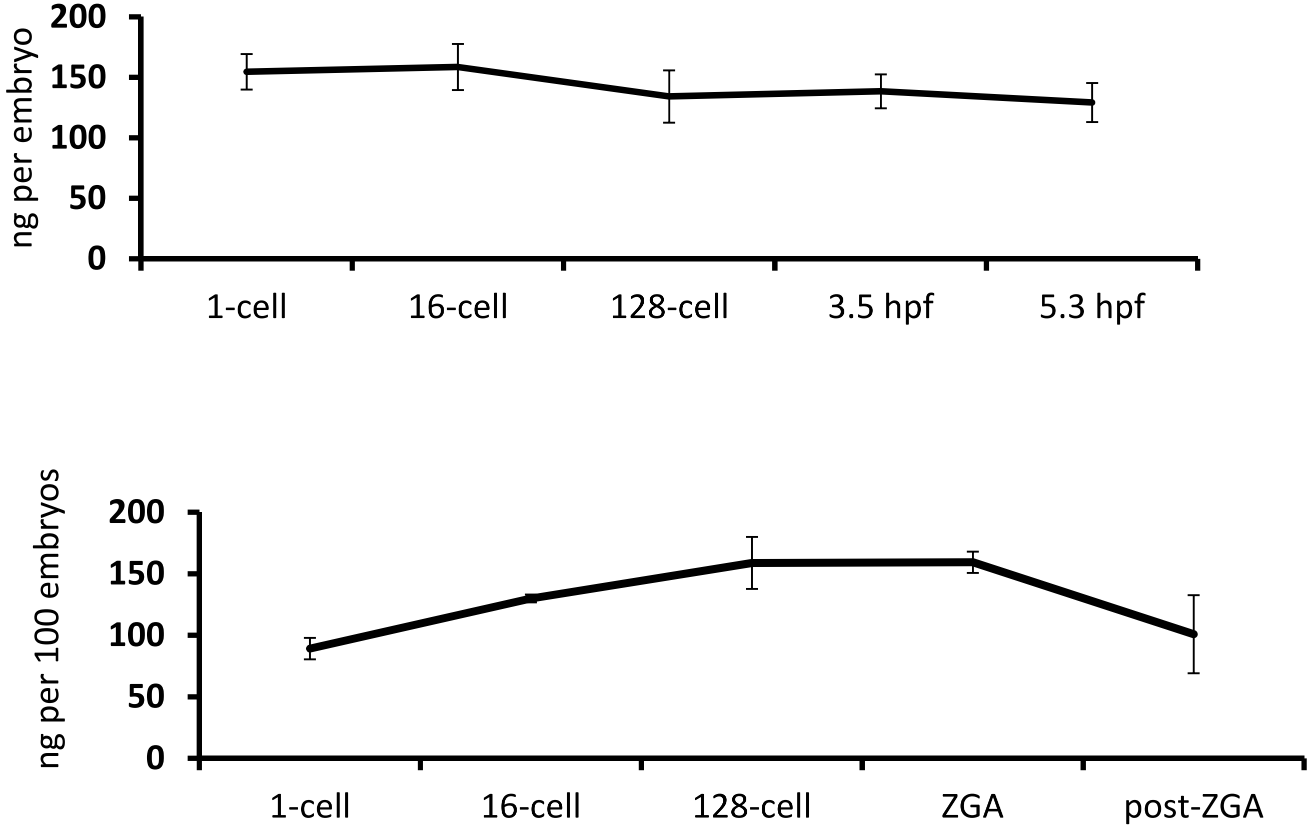

200
150
100
50
0
ng per embryo
1-cell
16-cell
128-cell
3.5 hpf
5.3 hpf
200
150
100
50
0
ng per 100 embryos
1-cell
16-cell
128-cell
ZGA
post-ZGA

Fig. S2

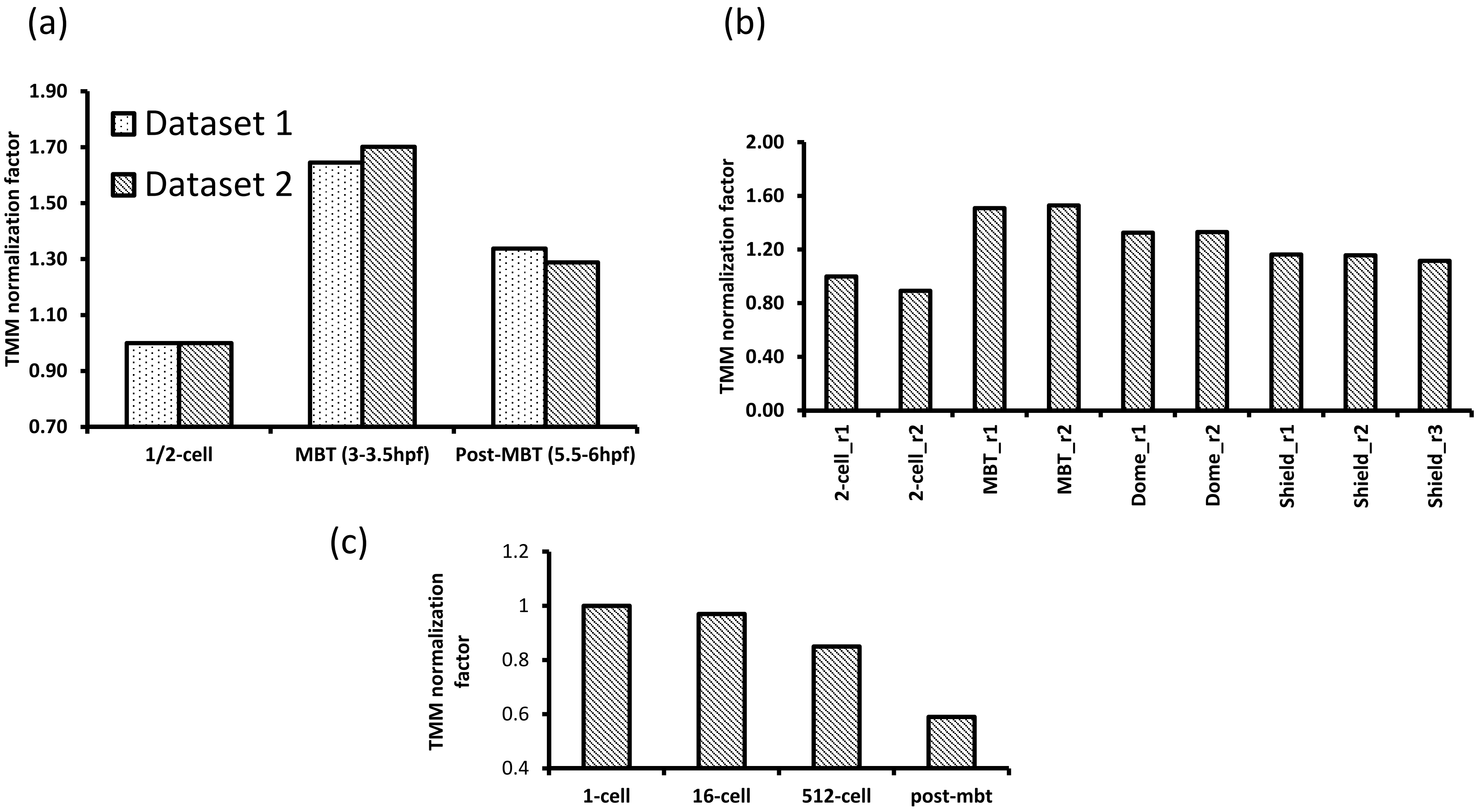
(a)
Dataset 1
Dataset 2
TMM normalization factor
1.90
1.70
1.50
1.30
1.10
0.90
0.70
1/2-cell
MBT (3-3.5hpf)
Post-MBT (5.5-6hpf)
(b)
TMM normalization factor
2.00
1.60
1.20
0.80
0.40
0.00
2-cell_r1
2-cell_r2
MBT_r1
MBT_r2
Dome_r1
Dome_r2
Shield_r1
Shield_r2
Shield_r3
(c)
TMM normalization factor
1.2
1
0.8
0.6
0.4
1-cell
16-cell
512-cell
post-mbt

Fig. S3

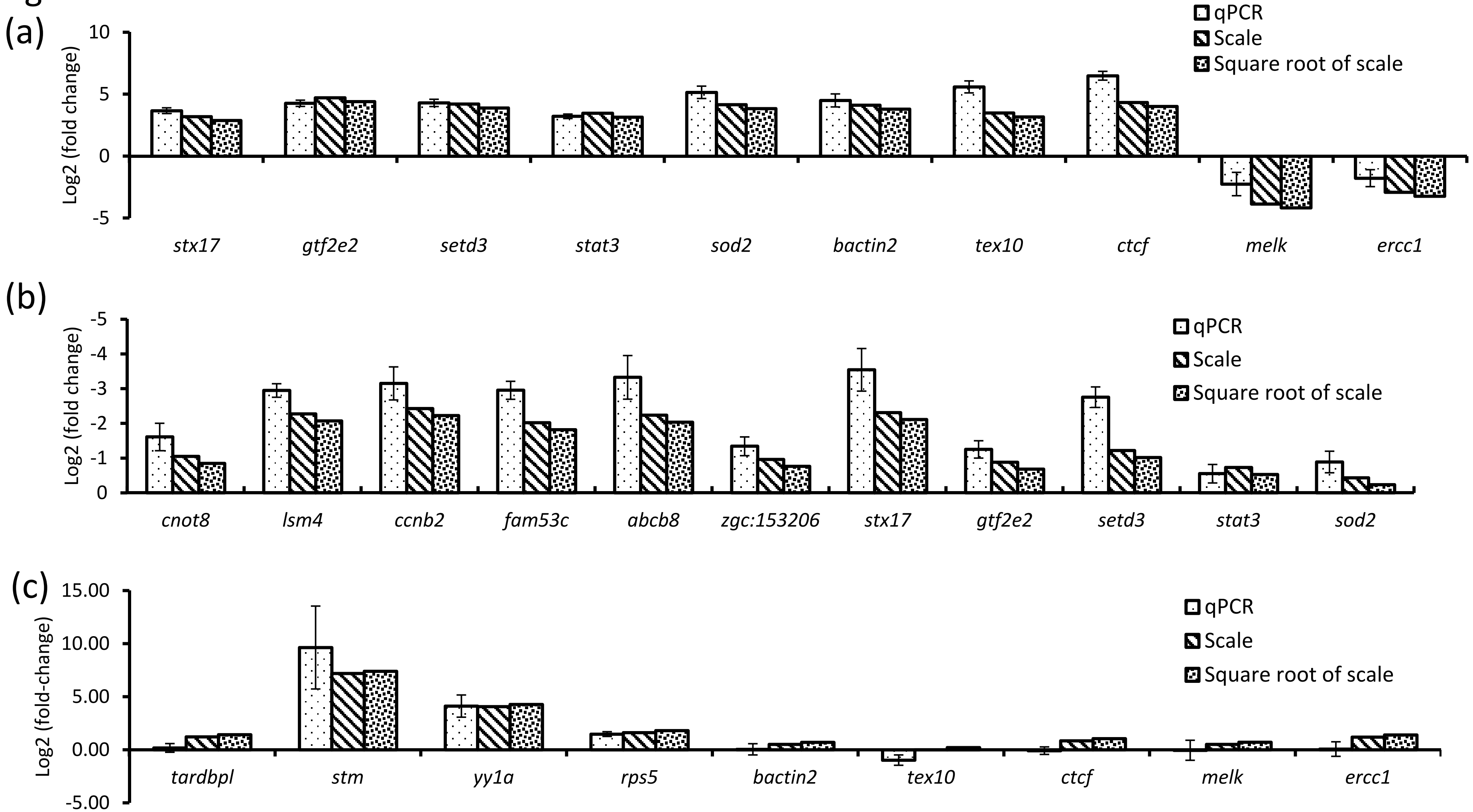

(a)
qPCR
Scale
Square root of scale
Log2 (fold change)
10
5
0
-5
stx17
gtf2e2
setd3
stat3
sod2
bactin2
tex10
ctcf
melk
ercc1
(b)
qPCR
Scale
Square root of scale
Log2 (fold change)
-5
-4
-3
-2
-1
0
cnot8
lsm4
ccnb2
fam53c
abcb8
zgc:153206
stx17
gtf2e2
setd3
stat3
sod2
(c)
qPCR
Scale
Square root of scale
Log2 (fold-change)
15.00
10.00
5.00
0.00
-5.00
tardbpl
stm
yy1a
rps5
bactin2
tex10
ctcf
melk
ercc1

Fig. S4

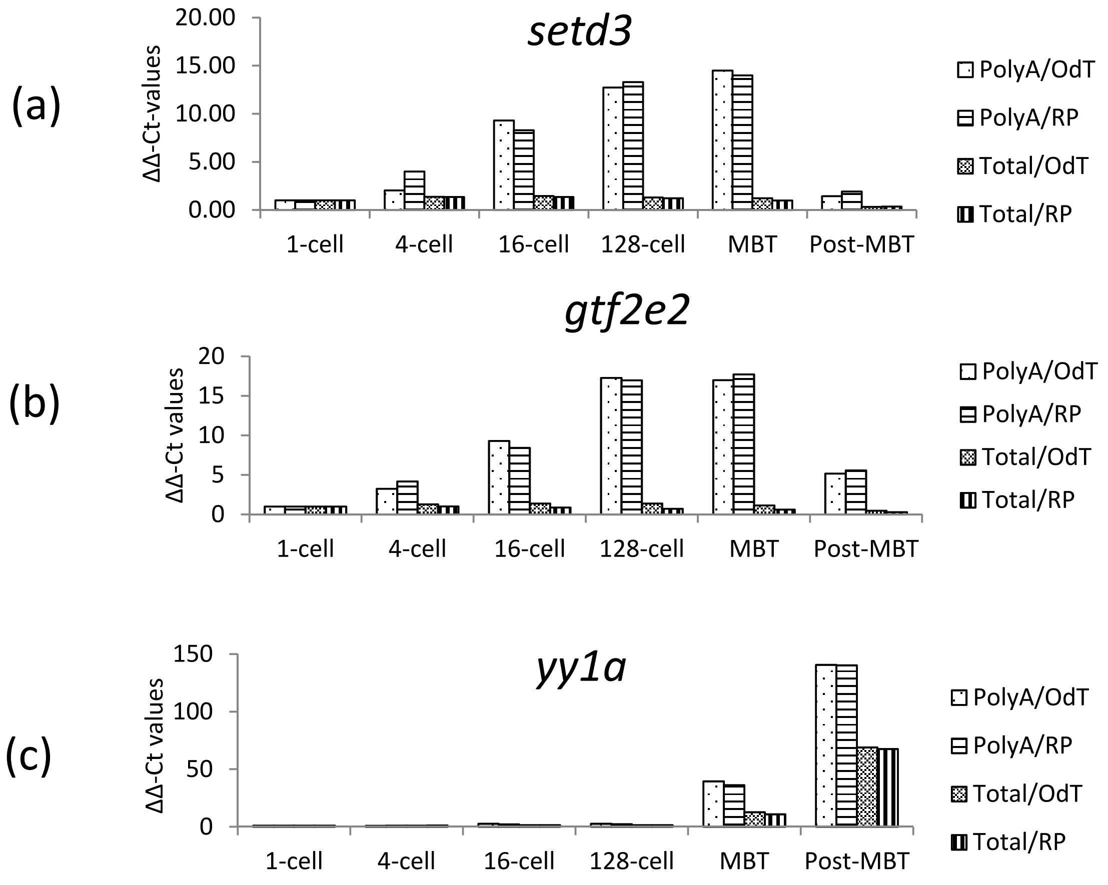

(a)
setd3
ΔΔ-Ct-values
20.00
15.00
10.00
5.00
0.00
1-cell
4-cell
16-cell
128-cell
MBT
Post-MBT
PolyA/OdT
PolyA/RP
Total/OdT
Total/RP
(b)
gtf2e2
ΔΔ-Ct values
20
15
10
5
0
1-cell
4-cell
16-cell
128-cell
MBT
Post-MBT
PolyA/OdT
PolyA/RP
Total/OdT
Total/RP
(c)
yy1a
ΔΔ-Ct values
150
100
50
0
1-cell
4-cell
16-cell
128-cell
MBT
Post-MBT
PolyA/OdT
PolyA/RP
Total/OdT
Total/RP

Fig. S5

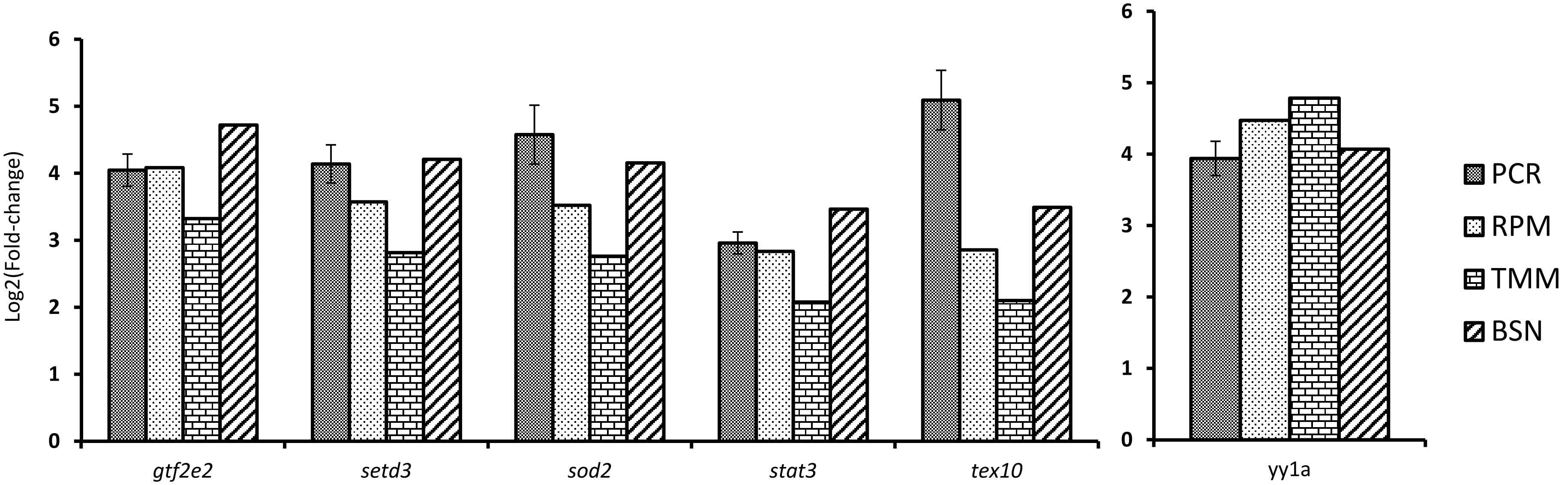

6
5
4
3
2
1
0
Log2(Fold-change)
gtf2e2
setd3
sod2
stat3
tex10
yy1a
PCR
RPM
TMM
BSN

Fig.S6

(a)

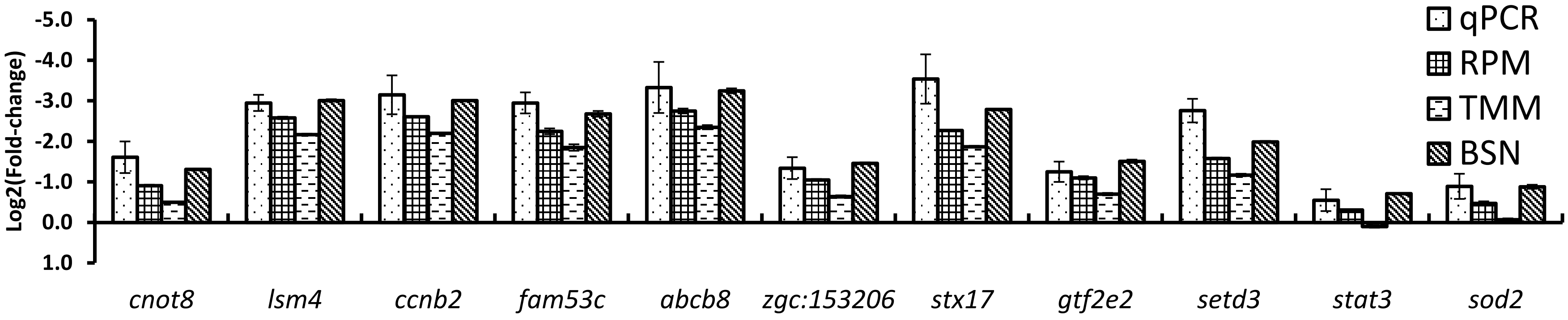

-5.0
-4.0
-3.0
-2.0
-1.0
0.0
1.0
Log2(Fold-change)
qPCR
RPM
TMM
BSN
cnot8
lsm4
ccnb2
fam53c
abcb8
zgc:153206
stx17
gtf2e2
setd3
stat3
sod2

(b)

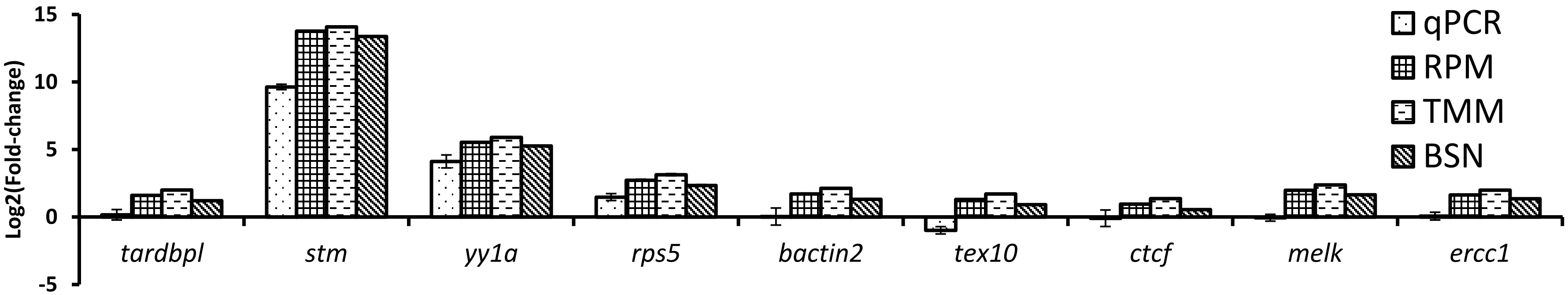

15
10
5
0
-5
Log2(Fold-change)
qPCR
RPM
TMM
BSN
tardbpl
stm
yy1a
rps5
bactin2
tex10
ctcf
melk
ercc1
